# Bacteriomic Profiles of Rock-Dwelling Lichens from the Venezuelan Guiana Shield and the South African Highveld Plateau

**DOI:** 10.3390/microorganisms12020290

**Published:** 2024-01-29

**Authors:** Zichen He, Takeshi Naganuma, Haemish I. A. S. Melville

**Affiliations:** 1 Graduate School of Integrated Science for Life, Hiroshima University, 1-4-4 Kagamiyama, Higashi-hiroshima 739-8528, Japan; 2 Department of Environmental Sciences, College of Agriculture and Environmental Sciences, University of South Africa, Florida, 0-41 Calabash Building, Unisa Science Campus, cnr Pioneer Avenue and Christiaan de Wet Road, Florida 1710, Gauteng, South Africa; melviha@unisa.ac.za

**Keywords:** V3–V4 region, 16S rDNA, MiSeq, OTUs, biogeography, metabolism, host-associated bacteria

## Abstract

Lichens are not only fungal–algal symbiotic associations but also matrices for association with bacteria, and the bacterial diversity linked to lichens has been receiving more attention in studies. This study compares the diversity and possible metabolism of lichen-associated bacteria from saxicolous foliose and fruticose taxa *Alectoria*, *Canoparmelia*, *Crocodia*, *Menegazzia*, *Usnea*, and *Xanthoparmelia* from the Venezuelan Guiana Shield and the South African Highveld Plateau. We used DNA extractions from the lichen thalli to amplify the eukaryotic 18S rRNA gene (rDNA) and the V3–V4 region of the bacterial 16S rDNA, of which amplicons were then Sanger- and MiSeq-sequenced, respectively. The V3–V4 sequences of the associated bacteria were grouped into operational taxonomic units (OTUs) ascribed to twelve bacterial phyla previously found in the rock tripe *Umbilicaria* lichens. The bacterial OTUs emphasized the uniqueness of each region, while, at the species and higher ranks, the regional microbiomes were shown to be somewhat similar. Nevertheless, regional biomarker OTUs were screened to predict relevant metabolic pathways, which implicated different regional metabolic features.

## 1. Introduction

Lichens are the symbiotic associations of fungal mycobionts and algal/cyanobacterial photobionts and play essential ecological roles in many ecosystems. They can be primary colonizers of bare rock, soil, or wood, initiating the process of soil formation and nutrient cycling. They can also contribute to ecosystems via nitrogen fixation when cyanobacteria participate in symbiosis and serve as food sources for various animals. In addition, lichens are indicators of air pollution, climate change, and a variety of other environmental changes [1,2].

As pioneer organisms, lichens are often the first to colonize bare rocks. They can do this because they are able to survive in extreme environments such as deserts, tundra, and bare rock surfaces [3]. Of the estimated 5 million fungal species [4], only 156,287 have been included in the Species Fungorum (as of 12 December 2023) [5], and 19,387 are lichen-forming species [6], of which ca. 10–20% are regarded as rock-dwelling or epilithic lichens [1,7,8].

Bacterial communities associated with lichens have been studied from the holobiont viewpoint [9], especially with the advent of multi-omics and high-throughput sequencing techniques [10,11]. Studies on lichen-associated microbiomes often include or target epilithic lichens and report the bacterial families of *Acetobacteraceae*, *Acidobacteriaceae* and *Actinomycetaceae* as well as the alphaproteobacterial families of *Acetobacteraceae*, *Beijerinckiaceae*, *Brucellaceae*, *Methylobacteriaceae*, and *Sphingomonadaceae* [12,13,14,15,16,17,18,19,20,21]. In this study, data from the analysis of epilithic lichens, collected from rocks or cliffs in highlands in the Venezuelan Guiana Shield and the South African Highveld Plateau, are presented.

This study presents the V3–V4 region of the 16S rDNA-based or amplicon-based microbiomes of 20 samples of epilithic lichens of the genera *Alectoria*, *Canoparmelia*, *Menegazzia*, and *Usnea,* which belong to the family *Parmeliaceae,* and *Crocodia,* which belongs to the subfamily *Lobarioideae* (Lumbsch and S.D.Leav.) of the family *Peltigeraceae* [22]. These include *Parmeliaceae*, which is the most predominant family of lichen-forming fungi and of epilithic lichens [6], and *Peltigeraceae*, which contains tripartite lichens [23]. The bacterial microbiomes associated with these families, including cyanobacteria, are compared among lichen taxa and between the two geographically distinct highland regions, i.e., the Guiana Shield and the South African Highveld Plateau. Possible impacts of lichen taxa and geographical settings on the lichen-associated bacterial microbiomes are evaluated.

## 2. Materials and Methods

### 2.1. Collection of Epilithic Lichen Samples

**Figure 1:**
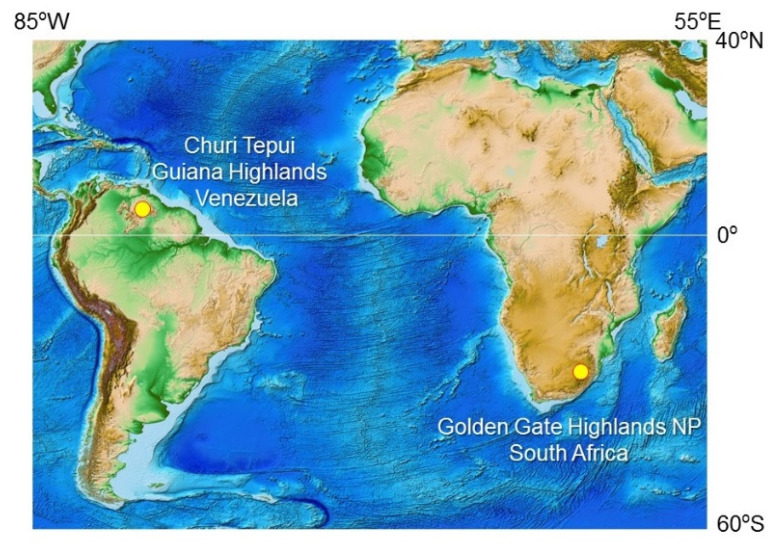
Sampling sites of epilithic lichens at Churi Tepui in the Venezuelan Guiana Shield and the Golden Gate Highlands National Park in the South African Highveld Plateau. The ETOPO1 Global Relief Model [30] is the source of the map image.

The lichen samples of the Venezuelan Guiana Shield were collected in October 2016 on the summit of the table-top mountain or tepui called *Churi* (ca. 05°15′ N, 62°00′ W; Figure 1, Table 1) during the speleological expedition to the tepui’s cave system [24]. Tepuis are typically flat-topped mountains with steep vertical walls rising to 1000 m or more above the surrounding landscape, and with an annual rainfall >3000 mm [25]. Due to their elevation and isolation, they create their own microclimates, with cooler temperatures and higher humidity than the surrounding lowlands. The temperature on the tepui summits can vary depending on the time of day and season. During the day, temperatures can range from 10 °C to 20 °C, with temperatures being cooler at higher elevations. At night, temperatures can drop to 0 °C (or lower), depending on the season and elevation [26,27].

The lichen samples from the South African Highveld Plateau were collected in October 2018 from rocks and cliffs in grassland and bush along a stream in the Golden Gate Highlands National Park (ca. 28°52′ S, 28°60′ E; Figure 1, Table 1). The park is situated in the foothills of the Maluti Mountains and is characterized by high-altitude grasslands, rolling hills, valleys, and sandstone cliffs. The park has a high-altitude climate, with cool temperatures and low humidity. The average temperature ranges from 13 °C to 26 °C in summer and from 1 °C to 15 °C in winter. Rainfall is concentrated in the summer months from November to February, with an average annual rainfall of approximately 650 mm to 760 mm [28,29].

All Guiana Shield lichens (G01 to G12) and half of the South African lichens (SA01, SA03, SA05 and SA07) were collected from bare rocks in grassland, but the other half (SA02, SA04, SA06 and SA08) were collected from bare rocks in bush. Thalli of epilithic lichens were cut with a flamed field knife and put into Whirl-Pak bags (Nasco, Fort Atkinson, WI, USA). The lichen thalli samples were air-dried, stored in the dark on site, transferred to the laboratory at Hiroshima University, and frozen at −25 °C in preparation for bulk DNA extraction.

### 2.2. Bulk DNA Extraction from Lichen Thalli

Thalli of a lichen sample were cleaned with autoclaved Milli-Q ultrapure water, cut into pieces, ground to finer fragments, and homogenized, one gram of which was used for DNA extraction by the method detailed in previous studies [20,21]. Although they were not examined under a microscope, cephalodia were not clearly visible. The extracted DNA was maintained at −20 °C until PCR amplification.

### 2.3. Amplification and Sequencing of Fungal/Algal 18S rDNA

Near-full-length 18S rDNA of lichen-forming fungi and algae were amplified on two TaKaRa Thermal Cyclers with the primer sets shown in Table 2 and using the thermal cycling described in the previous study [21]. The PCR amplicons were purified and Sanger-sequenced at the Department of Gene Science, Natural Science Center for Basic Research and Development (N-BARD), Hiroshima University [21].

### 2.4. Amplification and Sequencing of V3–V4 Region of Bacterial 16S rDNA

Using the same prepared DNA, PCR amplification of the V3–V4 region of 16S rDNA was carried out with the specific primers 341F and 806R (Table 2). The thermal cycling conditions for the PCR were as follows: 95 °C for 3 min; 25 cycles of 95 °C for 30 s, 55 °C for 30 s, and 72 °C for 30 s; and finally, 72 °C for 5 min.

To construct the sequence library and conduct paired-end 300 bp sequencing, the molecular diagnostic company, Environmental Research and Solutions Co. Ltd. (Kyoto, Japan), was employed [21].

### 2.5. Sequence Data Analysis and OTU Determination

The 18S rDNA sequences produced by Sanger sequencing were aligned using ClustalW and BioEdit to remove poor-quality sequences [31,32]. Then, the remaining sequences were manually assembled and checked for chimeras using tree topology analysis [33]. Finally, the resulting sequences were searched using BLAST to identify the lichen-forming fungi and algae.

The V3–V4 reads generated by MiSeq were processed using the microbiome taxonomic profiling (MTP) pipeline for EzBioCloud (https://www.ezbiocloud.net/contents/16smtp; accessed on 12 December 2023) [34]. Any unclear reads (with less than 100 nucleotides or low average quality scores <25) were excluded from the analysis [21]. After removing any duplicated sequences, the unique reads were compared with the 16S rDNA sequence database PKSSU4.0 of EzBioCloud.

The taxonomic classification was carried out by comparing the sequence similarity of reads to reference sequences, where the similarity cutoffs were defined as follows: ≥97%, <97–94.5%, <94.5–86.5%, <86.5–82%, <82–78.5%, and <78.5–75% for species, genus, family, order, class, and phylum, respectively [35], with any reads below these cutoffs labeled as unclassified and marked with the suffix “_uc”. Any unidentified reads at the species level (with less than 97% similarity) were subjected to chimera-checking using the chimera-free reference database at EzBioCloud (https://help.ezbiocloud.net/mtp-pipeline/; accessed on 12 December 2023). Any chimera, singleton, and eukaryotic plastid reads were excluded from further analysis. The remaining V3–V4 sequences were then grouped into operational taxonomic units (OTUs) with a 97% cutoff value [34], and the representative OTUs were subjected to BLAST searching.

The sequences of 18S rDNA for lichen-forming fungi and algae have been deposited in the DDBJ/ENA/GenBank database with accession numbers ranging from LC761218 to LC761237 and LC761244 to LC761263, respectively. The V3–V4 reads are accessible at the DDBJ Sequence Read Archive (DRA) and can be found under the accession numberDRA015994. The associated BioProject and BioSample numbers are PRJDB15406 and SAMD00585845 to SAMD00585864, respectively, and the sample-to-number correspondence can be found in Table S1.

### 2.6. Diversity Indices and Bioinformatic Analyses of OTUs

The MTP pipeline of EzBioCloud was used to analyze the rarefaction curves. Specifically, using the alpha diversity indices (Chao1, Shannon, and Simpson indices), the richness and evenness of bacterial OTUs associated with the lichen samples were estimated by EzBioCloud MTP. Chao1 corresponds to a rarefaction curve asymptote as an estimator of species richness or OTU richness [36]. Shannon and Simpson indices were also used to calculate an “effective number of species” (*ENS*) [37] or an effective number of OTUs of a sample. It is important to note that the Chao1 index takes singletons into account.

For beta diversity, the OTUs were clustered based on the UniFrac distance matrix [38], and biomarker OTUs were screened using the linear discriminant analysis (LDA) [39] and LDA–effect size method (LEfSe) [40] with LDA scores of 4.0 and 4.5 as the thresholds to screen, respectively. Differential abundance was analyzed using the analysis of compositions of microbiomes with bias correction (ANCOM-BC) [41].

The biomarker OTUs at species rank with LDA scores > 4.0 were projected on the metabolic pathways of the Kyoto Encyclopedia of Genes and Genomes (KEGG; http://www.genome.jp/kegg/; accessed on 12 December 2023) [42] using the VANTED (version 2.8.8; https://www.cls.uni-konstanz.de/software/vanted/; accessed on 12 December 2023) [43] and the PICUSt 2.0 programs [44].

## 3. Results

### 3.1. Identification of Mycobionts and Photobionts of the Epilithic Lichens

All of the mycobionts of the epilithic lichens from the Guiana Shield and Highveld Plateau were attributed to genera of the class *Lecanoromycetes* (Table 3), the largest class among the lichen-forming fungi [45]. Similarity values were at least 98.58% (Table S2). The samples G07–G12 from the Guiana Shield and SA01–SA02 from the South African Highveld Plateau were affiliated with the same lichen species, *Canoparmelia caroliniana* (Nyl.) Elix and Hale, known to occur in the Americas and East Africa [46]. The other samples were affiliated with different species by region. Fruticose lichens from the Guiana Shield were affiliated with the species *Alectoria sarmentosa* (Ach.) Ach. (G01, G02) and *Usnea florida* (L.) Weber ex F.H.Wigg. (G03, G04). The samples G05 and G06 were affiliated with *Menegazzia terebrata* (Hoffm.) A.Massal., a sub-cosmopolitan (excluding Antarctica) lichen [47]. The South African samples SA03-SA05 were affiliated with sub-cosmopolitan species *Xanthoparmelia conspersa* (Ehrh. ex Ach.) Hale [48]. The samples SA06–SA08 were affiliated with the only peltigeracean species in this study, *Crocodia aurata* (Ach.) Link (also a cosmopolitan species) [49].

All of the algal photobionts were most closely related to the green algal species ascribed to the genus *Trebouxia*, the most prevalent photobiont among lichens [50]. Similarity values were 98.44% or higher (Table S3). However, paraphyly in the genus *Trebouxia* and delineation of new genera *Asterochloris* and *Vulcanochloris* leaves room for considering members of the new genera as photobionts of the studied lichens [51,52].

*Crocodia aurata* (SA06–SA08) has two photobionts of a cyanobacterial *Nostoc* species and a green algal species of the genus *Dictyochloropsis* (family *Trebouxiaceae*). However, *C. aurata* contained *Trebouxia aggregata* (Archibald) Gärtner (in SA06 and SA07) or *Trebouxia* sp. SAG2463 (in SA08) and *Nostoc* or other cyanobacteria but at <1% of total bacterial OTUs (Table S4).

Cyanobacterial OTUs were present in all of the samples. A total of 107 cyanobacterial OTUs were detected, of which 82 were affiliated with species or species-rank taxa (Table S4). The ratio of cyanobacterial reads to the total read number of a sample ranged from 0.11% in G01 with 5 OTUs to 13.26% and 11.01% in G05 and G04 with 28 and 19 OTUs, respectively. The highest ratio for a single OTU was 5.80% of “PAC002560_g_uc” (genus-rank, uncultured, details unknown) in G12, followed by 5.31% of “JN023297_s” (species-rank) [53] in G04. The same OTU “JN023297_s” was present at a ratio as high as 2.51% in G04 and in other samples at <1%. Other OTUs occurring at >1% were “PAC000112_s” in G07 and G11 at 3.09% and 3.06%, respectively; “DQ914863_g_uc” [54] in G05 and G08 at 2.17% and 1.10%, respectively; “FJ465967_s” [55] in G05 at 2.14%; “*Stigonema ocellatum*” in G05 and G03 at 1.41% and 1.16%, respectively; and “AY326529_s” [56] in G06 at 1.10%. The OTU affiliated with *Stigonema ocellatum* was also detected in the Antarctic epilithic lichen *Umbilicaria* [20]. The cyanobacteria represented by the OTUs with read frequencies > 1% may function as additional photobionts in tripartite lichens or multipartite lichens, as in the case of G05 with its two > 1% cyanobacterial OTUs; however, inclusion of cyanobacteria that are not associated with lichens but are nearby may not be ruled out (discussed later).

### 3.2. MiSeq-Generated V3–V4 Sequences and OTUs

A total of 1,010,008 raw reads from the 20 lichen samples were generated using Illumina MiSeq sequencing, which were then filtered to 848,814 valid paired reads to be grouped into OTUs. Based on the analysis records in the EzBioCloud database [34], the mean length of all valid reads was 403.8 bp. For Guiana and South Africa, the mean length of valid reads was 401.8 bp and 406.8 bp, respectively.

Rarefaction curves were generated using the read and OTU counts (Figure S1). The coverage of rarefaction analysis expressed the ratio of obtained OTUs (Table 4) against the estimated total OTUs (a rarefaction asymptote in Figure S1), the latter of which is equivalent to the alpha diversity index, Chao1 (shown later). The mean, minimum, and maximum coverage ratios were 95.42%, 87.24% (in G02), and 99.67% (in G06), respectively. The coverage ratios suggest that the valid reads generated in this study are sufficient for further statistical and bioinformatic analyses.

Table 5 shows the regional numbers of taxa (OTU, species, genus, family, order, class, and phylum) detected only in the Guiana lichen samples, the South African samples, and both regions’ samples. The observed bacterial OTUs showed higher percentages of region-specific features. However, regional traits were somewhat ambiguous at the species and higher ranks, with the regions’ common OTUs being more than half of total OTUs at the order, class, and phylum ranks. The results emphasize the uniqueness of each region at the OTU rank and the similarity of the two areas at higher ranks.

### 3.3. Taxonomic Composition of Lichen-Associated Bacterial Community

**Figure 2:**
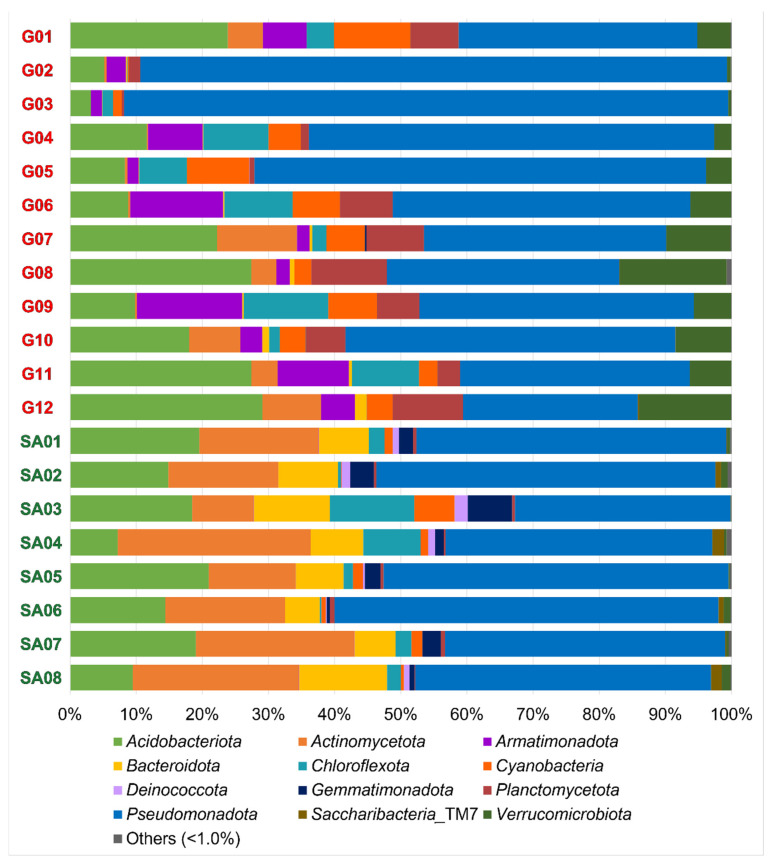
Compositions of the OTU-derived bacterial phyla of lichens from the Venezuelan Guiana Shield (G01 to G12) and the South African Highveld Plateau (SA01 to SA08). Twelve phyla were observed with >1% read abundances. Compositions of the OTU-derived bacterial classes, orders, families, and genera are shown in Figures S2–S5.

Compositions of the OTU-derived bacterial phyla in 20 lichen samples are shown in Figure 2. A total of 12 bacterial phyla are portrayed as the standard features in all 20 samples. Each lichen sample contained 10 to 23 bacterial phyla (Table 4), including the 4–13 phyla consisting of less than 1% (of total) reads in each sample. The most common were *Acidobacteriota*, *Actinomycota*, *Armatimonadota*, *Bacteroidota*, *Chloroflexota*, *Cyanobacteria*, *Deinococcota*, *Gemmatimonadota*, *Planctomycetota*, *Pseudomonadota*, *Saccharibacteria*_TM7 and *Verrucomicrobiota*.

At the family level, the overall top five families in this study were acidobacterial *Acidobacteriaceae* and *Bryobacteraceae*, and alphaproteobacterial *Acetobacteraceae*, *Beijerinckiaceae*, and *Sphingomonadaceae* (Figure S4). These are compared with microbiomes of Thai tropical lichens, whose top five families are *Beijerinckiaceae*, *Chthoniobacteraceae* (phylum *Verrucomicrobiota*), *Acetobacteraceae*, *Gemmataceae* (phylum *Planctomycetota*), and an unidentified family in the order *Tepidisphaerales* (phylum *Planctomycetota*) [57]. The difference in the top five families can be ascribed to biogeography as well as to host lichen species. Of ten Thai lichens, three and one belong to the families *Parmeliaceae* and *Peltigeraceae*, respectively, but all belong to genera that are different from those in this study.

### 3.4. Alpha and Beta Diversity

Alpha diversity indices, Chao1, Shannon, and Simpson (Table 6), were used to calculate the effective number for species (*ENS*) [37]. Chao1, Shannon, *ENS* values and observed OTU numbers have positive correlations with species and evenness, and Simpson index values negatively correlate with species and evenness. Therefore, higher Chao1, Shannon, *ENS* values, observed OTU numbers, and lower Simpson index values found in South African samples indicate higher species richness and evenness.

Due to different calculation methods, Shannon and Simpson indices could not be used to estimate the richness of bacterial species. Comparatively, Chao1 values were close to estimated OTU numbers and may better represent species richness of a large sample size, as reported in other studies [25,58].

**Figure 3:**
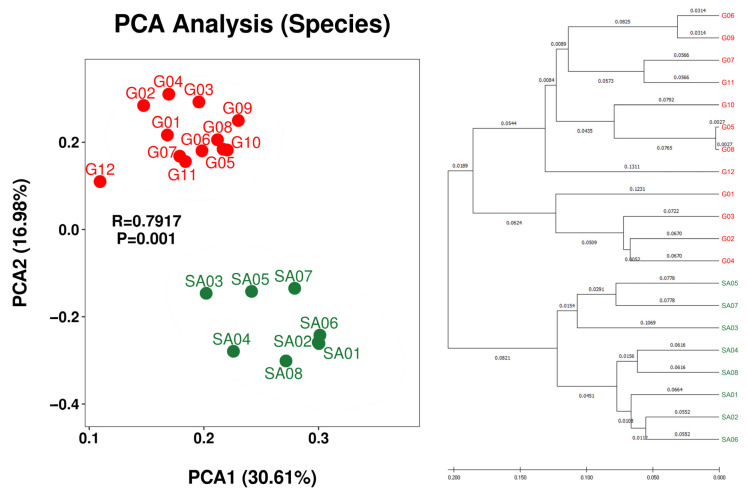
PCA plot (**left**) and hierarchical clustering dendrogram (**right**) of OTU-derived bacterial species of the lichens from the Venezuelan Guiana Shield (red) and the South African Highveld Plateau (green). PCA plots at higher taxa (genus, family, order, class, and phylum) are shown in Figure S6.

Beta diversity analysis demonstrated regional separation between the Guiana Shield (G01 to G12) and South Africa (SA01 to SA08) at the species rank (Figure 3) and the genus and class ranks (Figure S6). Regional separation is unclear at the family, order, and phylum ranks (Figure S6), which may be influenced by the “common” taxa at these ranks between the two regions (Table 5). Weak intra-regional separation or intra-regional variation between the bush samples (SA02, SA04, SA06 and SA08) and the grassland samples (SA03, SA05 and SA07) is implied; however, grouping of grassland SA01 with bush samples obscures the intra-regional variation. Nevertheless, monospecific intra-regional variation is also seen in the Guiana Shield samples, such as those between G05 and G06 of *Menegazzia terebrata* as well as those between G07 and G12 of *Canoparmelia caroliniana* (Figure 2 and Figure 3).

**Figure 4:**
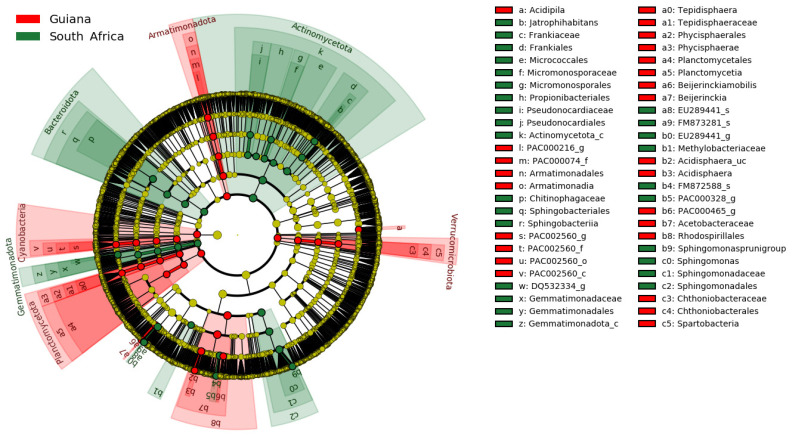
Microbiomic biomarkers of bacteria associated with the lichens from the Venezuelan Guiana Shield (red) and the South African Highveld Plateau (green), as shown in the cladogram generated by LEfSe [40]. The concentrically arranged nodes correspond to the domain bacteria, phylum, class, order, family, genus, and species from innermost to outermost. Red and green nodes/shades indicate significantly higher relative abundances of taxa. The diameters of the node circles are proportional to the abundance of corresponding taxa.

The regional distinctions of OTUs are presented in the phylogenetic cladogram (Figure 4). Substantial biomarkers, listed in Table 7, were selected by setting the LDA score threshold to 4.5. Substantial biomarkers for the Venezuelan Guiana Shield included two taxa, the family *Acetobacteraceae* and the order *Rhodospirillales*, both affiliated with the phylum *Pseudomonadota*. Biomarkers for the South Africa Highveld Plateau included seven taxa: the genus *Sphingomonas*, the family *Sphingomonadaceae*, the order *Sphingomonadales*, the genus-raked EU289441_g of the family *Beijerinckiaceae*, and the order *Frankiales* affiliated with the class-rank *Actinomycetota*_c of the phylum *Actinomycetota*. No biomarkers at the species rank were identified, with a threshold value of 4.5.

**Figure 5:**
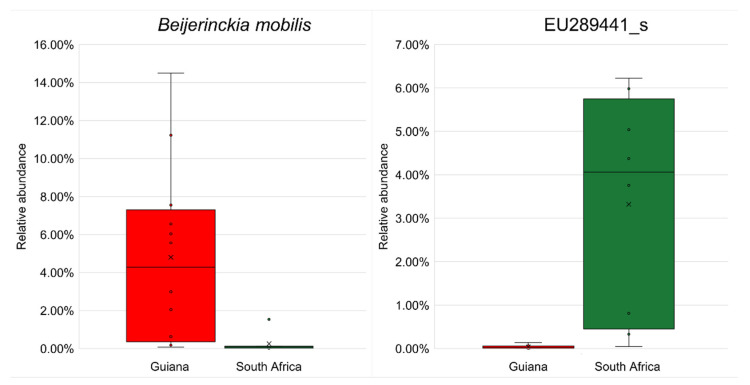
Significant differences (*p* < 0.05) in relative abundances of the most substantial biomarker OTUs from the Venezuelan Guiana Shield (red) and the South African Highveld Plateau (green) analyzed by ANCOM-BC. (**Left**) the most substantial biomarker for the Venezuelan Guiana Shield, *Beijerinckia mobilis* affiliated with the phylum *Pseudomonadota*. (**Right**) the most substantial biomarker for the South African Highveld Plateau, EU289441_s, affiliated with the genus *Lichenibacterium* of the same phylum *Pseudomonadota*. The bottoms and tops of boxes indicate the first and third quartiles, respectively; the bottoms and tops of whiskers indicate the 1.5 interquartile range beyond the lower and upper quartiles, respectively; the circles indicate the original data (including outliers); the crosses indicate the averages; and the horizontal lines indicate the medians. Other substantial biomarker OTUs are shown in Figure S7.

No biomarker OTU was identified at the species rank with an LDA score >4.5 (Table 7). However, by reducing the threshold to 4.0, six biomarkers, two from the Guiana Shield and four from the Highveld Plateau, were detected at the species rank and were subject to differential abundance analysis by ANCOM-BC. The most substantial biomarkers, nitrogen-fixing *Beijerinckia mobilis* (order *Hyphomicrobiales*, family *Beijerinckiaceae*) for Guiana Shield and EU289441_s affiliated with beta-carotene-producing *Lichenibacterium* (order *Hyphomicrobiales*, family *Lichenibacteriaceae* [59]) for South African Highveld Plateau, are shown in Figure 5, with other biomarkers shown in Figure S7.

Six biomarkers at species rank with LDA > 4 were further predicted for metabolic pathways of lichen-associated bacteria. At the KEGG Level 1, i.e., the highest metabolic categories on the KEGG database, these biomarker OTUs were related by relative abundances to the following five significant pathways: “Metabolism”, “Genetic information processing”, “Unclassified”, “Environmental information processing”, and “Cellular processes”. The "Metabolism" pathway showed the highest relative abundance, as high as over 50% in OTUs from both sampling regions (Figure S8).

At Level 2, i.e., sub-metabolic categories on the KEGG database, each biomarker OTU was related to the 25 pathways (Figure S9), of which the top five were carbohydrate metabolism (10.32% in the Guiana Shield samples and 10.71% in the South African samples), membrane transport (10.30% in Guiana and 11.13% in South Africa), amino acid metabolism (10.20% in Guiana and 10.58% in South Africa), replication and repair (7.55% in Guiana and 7.14% in South Africa) and energy metabolism (7.11% in Guiana and 6.39% in South Africa). The most substantial difference was identified in "membrane transport," which was more dominant in the South African OTUs. However, the greatest difference was only 0.83%.

**Figure 6:**
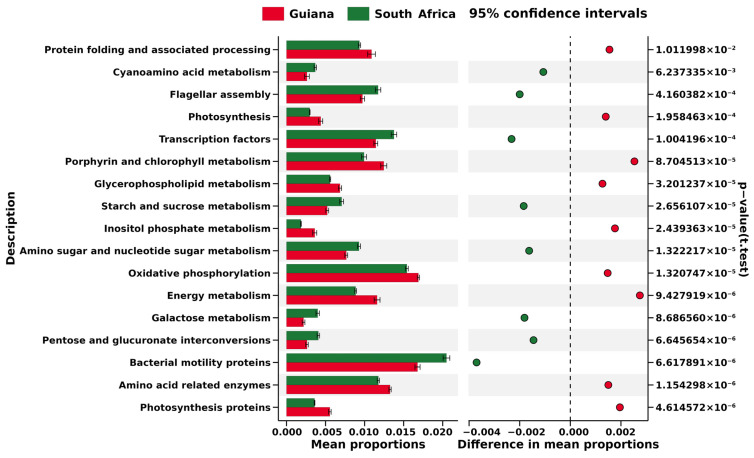
Excerpt of KEGG Level 3 pathways based on the biomarker OTUs of microbiomes associated with lichens from the Venezuelan Guiana Shield (red) and the South African Highveld Plateau (green). The horizontal axis indicates relative abundances (%) to be compared between the two regions. The pathways are screened by the cutoff values of >|0.0015| for the two regions’ mean relative abundance distances. Significant differences evaluated at *p* < 0.05 are indicated on the right side.

At Level 3, i.e., the most miniature metabolic category on the KEGG database, each biomarker OTU was related to 237 pathways (Figure S10), among which significant differences were identified in “transporters” (4.31% in Guiana and 4.91% in South Africa) and “bacterial motility proteins” (1.66% in Guiana and 2.04% in South Africa); however, the greatest difference was only 0.59%, identified in “transporters”. In addition, only 17 KEGG Level 3 metabolic pathways were found in the biomarker OTUs from Guiana (red) and South Africa (green), suggesting that metabolic pathways predicted from the two regions were different (Figure 6).

## 4. Discussion

Five and three species of epilithic lichens were sampled from the Venezuelan Guiana Shield and the South African Highveld Plateau, respectively. All of these species but one showed no overlaps between the regions, Carolina shield lichen (*Canoparmelia caroliniana*) was the only lichen species common to the two regions: six specimens, G07 to G12, from the Guiana Shield and two specimens, SA01 and SA02, from South Africa (Table 3). The associated bacterial microbiomes of the monospecific samples showed apparent regional features (Figure 3), implying that the associated microbiomes are controlled more regionally by climate, particularly by rainfall [24,25,26,27], than host lichen species. In contrast, the monospecific (*Canoparmelia caroliniana*) samples from grassland and bush, SA01 and SA02, respectively, showed no or little intra-regional variation (Figure 3), which may implicate a case of control by host species rather than inter-regional habitat variety in the vicinity. It is suggested that different alphaproteobacterial families of the order *Rhizobiales* (a synonym of *Hyphomicrobiales* [60]) are distributed in lichens [61], which is not yet well explained but may contribute to monospecific intra-regional variation. Lichen-Associated *Rhizobiales* 1 (LAR1) is a lineage of previously uncultured and most frequent non-cyanolichen-associates within the order *Rhizobiales* [13]. Nitrogen fixation was presumed as an eco-physiological function of LAR1 with which to rival cyanobacteria [13]; however, strains of *Lichenihabitans psoromatis*, the first cultured LAR1 species, possess no relevant genes in their genomes [62]. This study detected LAR1 only at <1% read abundance. Instead, the most abundant biomarker for Guiana Shield was affiliated with nitrogen-fixing *Beijerinckia mobilis* (Figure 5). Interplay among the hosts, green algal/cyanobacterial photobionts, LAR1, and other bacterial associates may influence “monospecific intra-regional variation” and would be a focus of the emerging multi-meta-omics [10].

Among the associated bacterial OTUs, cyanobacterial OTUs were present in all of the lichen samples at ratios ranging from 0.11% to 13.26% of the total OTUs (Table S4). High frequencies, >10%, were seen in *Usnea florida* (G04) and *Menegazzia terebrata* (G05) but not in samples of the same species, G03 and G06, respectively, indicating not species-related but site-related frequencies of cyanobacterial OTUs. Site-specific inclusion of not lichen-associated but nearby cyanobacteria may not be ruled out. *Crocodia aurata* (SA06 to SA08), potentially representing a cephalolichen, showed low frequencies, 0.46% to 1.62%, of cyanobacterial OTUs, implying little involvement of cephalodial cyanobacteria in the studied specimens.

The contribution of the cyanobacterial partner to the overall functioning of the lichen can vary depending on the species of the lichen and the specific ecological conditions. Although a technical threshold of 1% was set in this study, the eco-physiologically significant threshold for defining a cyanobacterium as a photobiont in a lichen must be specified. This can vary depending on the particular circumstances and the ecological importance of the cyanobacterium to the lichen community.

The photobiont biomass can range from less than 1% to greater than 90% of the total biomass of the lichen. This variation results from the different types of photobionts and their roles in symbiosis and from environmental factors such as light intensity, temperature, and water availability. Moreover, this biomass distribution can profoundly affect the morphology and ecology of lichens [1,63]. A recent comparison of the two sequencing methods revealed that most lichens have a single dominant photobiont genotype, which is representative of the vast majority of the thallus population [64]. Cyanobacteria occur in ca. 10% of the nearly 20,000 lichen species known as cyanolichens [65].

Cyanolichens are found in various terrestrial habitats, including tropical rainforests, semideserts, and arctic tundra. Their diversity and abundance are highest in humid environments [66]. Epiphytic species thrive in the moist and cool conditions of higher elevations in tropical mountains and maritime regions at higher latitudes. They are frequently abundant in the epiphyte communities of old-growth boreal and temperate forests, where they intercept and help retain atmospheric moisture, sequester nutrients, and provide habitat and food for numerous invertebrates [67]. Numerous epiphytic species flourish in microhabitats characterized by moderate light intensities, abundant moisture, and periodic drying events [68].

Tripartite cyanolichens host both green algal and cyanobacterial photobionts. In these lichens, the cyanobacteria, which are typically a minor component of the total photobiont biomass, are confined to structures known as cephalodia. Some green algal lichens frequently form ephemeral associations with free-living cyanobacteria, most likely to gain access to a fixed nitrogen source [24,69].

Field evidence implies that the identity of the *Nostoc* symbiont of bipartite and tripartite lichens depends on the degree of lichenization of the mycobiont than on the collection site [70]. In addition, the same *Nostoc* can be modified by the fungal host to function as the primary photobiont in a bipartite association or as a partner in a tripartite association [71]. The lichen-forming fungus can regulate the specific function of the cyanobacterium to optimize its fitness [72,73].

No cyanobacterial OTUs were screened as regional biomarkers. The (non-cyanobacterial) biomarkers were used to predict regional metabolic features such as “Energy metabolism”, “Porphyrin and chlorophyll metabolism”, and “Photosynthesis” for the Guiana Shield (Figure 6), which may be related to non-cyanobacterial photosynthetic activities, in addition to cyanobacterial photosynthesis, in the humid highland. In contrast, “Bacterial motility proteins” and “Flagellar assembly” were predicted for the South African Plateau (Figure 6), which may be linked to features that would help expand bacterial distribution and adhesion to a substrate in the dry highland. Geographical settings such as climate may thus have more impact on the microbiomes and relevant metabolisms associated with the epilithic lichens than host lichen species. This view, however, should be tested by comparing KEGG predictions derived from more diverse habitat features. Cellular functions, as well as gene expressions of bacterial associates, would also be viewed as an integral part of a meta-organism, lichen, rather than as the sum of responses of individual species [61].

## Figures and Tables

**Table 1:** Sampling sites of epilithic lichens inhabiting the rocks in the Venezuelan Chiuri Tepui and the South African Highveld Plateau. Coordinates (latitudes and longitudes) and elevations (altitudes) were determined with GPSMAP62S (Garmin, Olathe, KS, USA).

Region	Sample Code	Latitude	Longitude	Altitude
Churi Tepui, Guiana Shield, Venezuela	G01	05°15′11″ N to 05°15′13″ N	62°00′40″ W to 62°00′42″ W	2380 m to 2385 m
	G02			
	G03			
	G04			
	G05			
	G06			
	G07			
	G08			
	G08			
	G10			
	G11			
	G12			
Golden Gate National Park, Highveld Plateau, South Africa	SA01	28°30′03″ S	28°37′17″ E	2011 m
	SA02	28°30′04″ S	28°37′17″ E	2019 m
	SA03	28°29′34″ S	28°39′36″ E	2020 m
	SA04	28°30′04″ S	28°37′17″ E	2019 m
	SA05	28°29′22″ S	28°41′50″ E	1884 m
	SA06	28°30′04″ S	28°37′17″ E	2019 m
	SA07	28°30′05″ S	28°36′58″ E	1997 m
	SA08	28°30′04″ S	28°37′17″ E	2019 m

**Table 2:** Forward (F) and reverse (R) primers for PCR amplification of the target sequences.

Target Sequence	Primer Designation	F/R	Length (-mer)	5′ → 3′	Expected Product Size
Fungal 18S rDNA	NS17UCB	F	19	CATGTCTAAGTTTAAGCAA	2.0 kbp
	NS24UCB	R	20	AAACCTTGTTACGACTTTTA	
Algal 18S rDNA	Euk F	F	21	AACCTGGTTGATCCTGCCAGT	1.8 kbp
	Al1700r *	R	18	CTCCTTCCTCTAGGTGGG	
V3–V4 region of 16S rDNA	341F	F	17	CCTACGGGNGGCWGCAG	460 bp
	806R	R	21	GACTACHVGGGTATCTAATCC	

* Reverse-complement of Al1700f.

**Table 3:** Taxonomic classification and the closest species based on near-full-length 18S rDNA sequences of mycobiont fungi of the epilithic lichens from the Venezuelan Guiana Shield (G01 to G12) and the South African Highveld Plateau (SA01 to SA08). All of the listed taxa belong to the class *Lecanoromycetes*.

Sample Code	Closest Species				Common Name	Growth Form
	Order	Family	Genus	Species		
G01	*Lecanorales*	*Parmeliaceae*	*Alectoria*	*sarmentosa*	Witch’s hair lichen	Fruticose
G02						
G03			*Usnea*	*florida*	Beard lichen	
G04						
G05			*Menegazzia*	*terebrata*	Honeycombed lichen	Foliose
G06						
G07			*Canoparmelia*	*caroliniana*	Carolina shield lichen	
G08						
G09						
G10						
G11						
G12						
SA01						
SA02						
SA03			*Xanthoparmelia*	*conspersa*	Rock-shield lichen	
SA04						
SA05						
SA06	*Peltigerales*	*Peltigeraceae* subfamily *Lobarioideae*	*Crocodia*	*aurata*	Specklebelly lichen	
SA07						
SA08						

**Table 4:** Numbers of MiSeq-generated V3–V4 region reads, 97% similarity-based OTUs, OTU-derived species, genera, families, orders, classes, and phyla in each sample. Due to overlaps among samples, the subtotal and total numbers of taxa are smaller than the simple sums. Mean lengths of valid reads are also listed. Samples G01 to G12 were collected in the Venezuelan Guiana Shield, and samples A01 to A08 were collected in the South African Highveld Plateau.

Sample Code	Raw Read	Valid Read	OTU	Species	Genus	Family	Order	Class	Phylum	Mean Length (bp)
G01	38,482	33,984	326	249	119	62	42	27	12	396.5
G02	60,137	36,389	220	127	77	44	31	24	12	402.0
G03	39,256	32,219	392	195	115	58	39	29	12	400.3
G04	45,447	39,756	350	198	113	60	40	25	10	397.2
G05	100,000	83,914	1457	312	162	86	51	37	14	402.9
G06	37,458	35,926	800	566	224	94	59	38	15	402.0
G07	78,983	72,353	1202	536	222	90	58	39	17	407.4
G08	79,798	68,704	1141	309	176	89	53	38	14	402.5
G09	48,630	44,686	945	617	240	99	59	38	16	402.5
G10	37,557	36,431	669	386	164	77	51	33	14	398.7
G11	91,956	84,812	1666	782	273	113	71	44	19	406.4
G12	69,524	61,874	1104	507	218	93	58	38	16	402.6
Subtotal	727,228	631,048	3328	1399	547	204	106	60	23	401.8
SA01	28,046	22,570	1127	751	323	136	71	40	15	406.1
SA02	25,357	17,658	1074	750	305	134	69	38	17	406.9
SA03	65,414	53,190	1462	688	302	144	90	52	16	407.1
SA04	20,358	11,952	1204	909	392	157	78	46	19	407.3
SA05	38,753	32,312	1075	705	283	121	69	40	17	405.5
SA06	16,328	12,163	834	601	268	117	54	33	14	405.6
SA07	55,206	44,936	1644	1054	421	163	91	46	17	407.5
SA08	33,318	22,985	1423	893	378	148	74	41	15	408.1
Subtotal	282,780	217,766	3782	2221	755	275	133	67	23	406.8
Total	1,010,008	848,814	6051	2908	973	331	157	79	26	403.8

**Table 5:** Numbers of assigned OTUs and OTU-derived taxa (species, genera, families, orders, classes, and phyla) that were detected only in the Venezuelan Guiana Shield, only in the South African Highveld Plateau, and in both highland regions. The total numbers are the same as those in Table 4.

Distribution	Observed OTU	Species	Genus	Family	Order	Class	Phylum
Only in the Guiana region	2269	687	218	56	24	12	3
Only in the South Africa region	2723	1509	426	127	51	19	3
Common to both regions	1059	712	329	148	82	48	20
Total	6051	2908	973	331	157	79	26

**Table 6:** Alpha diversity indices (Chao1, Shannon, and Simpson) for the bacterial OTUs of 12 epilithic lichen samples from Venezuelan Guiana Shield (G01 to G12) and eight samples from South African Highveld Plateau (SA01 to SA08). The Shannon and Simpson indices calculated values of the effective numbers of species (*ENS*).

Sample Code	Observed OTU	Chao1	Shannon (*ENS*)		Simpson (*ENS*)	
G01	326	373.7	3.30	*27.1*	0.09	*11.1*
G02	220	229.6	1.57	*4.8*	0.49	*2.0*
G03	392	400.9	3.76	*42.9*	0.08	*12.5*
G04	350	363.4	3.31	*27.4*	0.14	*7.1*
G05	1457	1461.9	5.41	*223.6*	0.01	*100.0*
G06	800	846.8	5.09	*162.4*	0.01	*100.0*
G07	1202	1223.9	5.27	*194.4*	0.01	*100.0*
G08	1141	1147.3	5.27	*194.4*	0.01	*100.0*
G09	945	994.1	5.11	*165.7*	0.01	*100.0*
G10	669	689.0	5.19	*179.5*	0.01	*100.0*
G11	1666	1699.7	5.59	*267.7*	0.01	*100.0*
G12	1104	1117.5	4.98	*145.5*	0.02	*50.0*
**Average**	**856.0**	**879.0**	**4.49**	***136.3***	**0.07**	***65.2***
SA01	1127	1200.3	5.36	*212.7*	0.01	*100.0*
SA02	1074	1169.0	5.31	*202.4*	0.01	*100.0*
SA03	1462	1502.7	5.15	*172.4*	0.02	*50.0*
SA04	1204	1341.4	5.68	*292.9*	0.01	*100.0*
SA05	1075	1117.2	5.04	*154.5*	0.02	*50.0*
SA06	834	906.4	5.18	*177.7*	0.02	*50.0*
SA07	1644	1720.4	5.71	*301.9*	0.01	*100.0*
SA08	1423	1514.7	5.68	*292.9*	0.01	*100.0*
**Average**	**1230.4**	**1309.0**	**5.39**	***225.9***	**0.01**	***81.3***

**Table 7:** Biomarker OTUs and the corresponding taxa with the LDA scores > 4.5 identified in the lichen-associated microbiomes from the Venezuelan Guiana Shield and the South African Highveld Plateau.

Region	Code in Figure 4	Rank of Biomarker					LDA Score	*p*-Value
		Phylum	Class	Order	Family	Genus		
Venezuelan Guiana Shield	b8	*Pseudomonadota*	*Alphaproteobacteria*	*Rhodospirillales*			4.5925	0.025260
	b7	*Pseudomonadota*	*Alphaproteobacteria*	*Rhodospirillales*	*Acetobacteraceae*		4.5848	0.025260
South African Highveld Plateau	-	*Actinomycetota*					4.9213	0.000288
	k	*Actinomycetota*	*Actinomycetota*_c				4.9016	0.000213
	d	*Actinomycetota*	*Actinomycetota*_c	*Frankiales*			4.5269	0.000213
	b0	*Pseudomonadota*	*Alphaproteobacteria*	*Hyphomicrobiales* syn. *Rhizobiales*	*Lichenibacteriaceae*	EU289441_g	4.5234	0.013555
	c2	*Pseudomonadota*	*Alphaproteobacteria*	*Sphingomonadales*			4.6048	0.000213
	c1	*Pseudomonadota*	*Alphaproteobacteria*	*Sphingomonadales*	*Sphingomonadaceae*		4.6021	0.000288
	c0	*Pseudomonadota*	*Alphaproteobacteria*	*Sphingomonadales*	*Sphingomonadaceae*	*Sphingomonas*	4.5524	0.000517

## Data Availability

The raw sequence data, project data, and sample data of Guiana and South Africa regions are available at the DDBJ Sequence Read Archive (DRA015994), BioProject (PRJDB15406), and BioSample (SAMD00585845 to SAMD00585864), respectively.

## Supplementary Materials

The following supporting information can be downloaded at https://www.mdpi.com/article/10.3390/microorganisms12020290/s1. Table S1: List of DDBJ BioProject, Sequence Read Archive (DRA), and BioSample numbers; Table S2: List of fungal 18S rRNA gene sequences with their accession numbers; Table S3: List of algal 18S rRNA gene sequences with their accession numbers; Table S4: List of 107 cyanobacterial OTUs and their read number frequencies (%); Figure S1: Rarefaction curves based on the numbers of reads and OTUs from lichen samples; Figure S2: Bacterial class compositions of the OTUs obtained from lichen samples; Figure S3: Bacterial order compositions of the OTUs obtained from lichen samples; Figure S4: Bacterial family compositions of the OTUs obtained from lichen samples; Figure S5: Bacterial genus compositions of the OTUs obtained from lichen samples; Figure S6: PCA plots of OTU-derived species to phyla of the lichen samples; Figure S7: Significant differences in relative abundances of indicator OTUs by ANCOM-BC; Figure S8: KEGG Level 1 metabolic pathways found in the biomarker OTUs; Figure S9: KEGG Level 2 metabolic pathways found in the biomarker OTUs; and, Figure S10: KEGG Level 3 metabolic pathways found in the biomarker OTUs.
