# Supplementary material for: Bacteriomic Profiles of Rock-Dwelling Lichens from the Venezuelan Guiana Shield and the South African Highveld Plateau
Source: Microorganisms. 2024 Jan 29;12(2):290. doi: 10.3390/microorganisms12020290 (PMC10892498; doi:10.3390/microorganisms12020290)
Supplement: Supplementary file 1 [file microorganisms-12-00290-s001.zip › microorganisms-2807680-supplementary.pdf]

Supplementary Materials

Bacteriomic Profiles of Rock-dwelling Lichens from the Vene-zuelan Guiana Shield and the South African Highveld Plateau

by Zichen He, Takeshi Naganuma, and Haemish I.A.S. Melville

Table S1. List of DDBJ BioProject, Sequence Read Archive (DRA), and BioSample numbers.

Table S2. List of fungal 18S rRNA gene sequences with their accession numbers.

Table S3. List of algal 18S rRNA gene sequences with their accession numbers.

Table S4. List of 107 cyanobacterial OTUs and the read number frequencies (%).

Figure S1. Rarefaction curves based on the numbers of reads and OTUs from lichen samples.

Figure S2. Bacterial class compositions of the OTUs obtained from lichen samples.

Figure S3. Bacterial order compositions of the OTUs obtained from lichen samples.

Figure S4. Bacterial family compositions of the OTUs obtained from lichen samples.

Figure S5. Bacterial genus compositions of the OTUs obtained from lichen samples.

Figure S6. PCA plots of OTU-derived species to phyla of the lichen samples.

Figure S7. Significant differences in relative abundances of indicator OTUs by ANCOM-BC.

Figure S8. KEGG Level 1 metabolic pathways found in the biomarker OTUs.

Figure S9. KEGG Level 2 metabolic pathways found in the biomarker OTUs.

Figure S10. KEGG Level 3 metabolic pathways found in the biomarker OTUs.

Table S1. List of DDBJ BioProject number, Sequence Read Archive (DRA) accession number, and BioSample accession numbers of the V3-V4 sequence datasets registered to the public database of DDBJ.

Sample	BioProject #	DRA acc. #	BioSample acc. #
G01			SAMD00585845
G02			SAMD00585846
G03			SAMD00585847
G04			SAMD00585848
G05			SAMD00585849
G06			SAMD00585850
G07			SAMD00585851
G08			SAMD00585852
G09			SAMD00585853
G11	PRJDB15406	DRA015994	SAMD00585854
G12			SAMD00585855
G12			SAMD00585856
SA01			SAMD00585857
SA02			SAMD00585858
SA03			SAMD00585859
SA04			SAMD00585860
SA06			SAMD00585861
SA07			SAMD00585862
SA08			SAMD00585863

Table S2. List of the near-full-length fungal 18S rRNA gene sequences with their accession numbers of the studied epilithic lichen samples, and the most closely related species with respective accession numbers as well as similarity values.

Sample		Closest species		
Code	Accession #	Name	Accession #	Similarity (%)
G01	LC761218	*Alectoria sarmentosa*	AF140233	99.17
G02	LC761219			99.52
G03	LC761220	*Usnea florida*	AF117988	99.76
G04	LC761221			99.71
G05	LC761222	*Menegazzia terebrata*	AY584661	99.58
G06	LC761223			99.17
G07	LC761224	*Canoparmelia caroliniana*	AY584658	99.38
G08	LC761225			99.56
G09	LC761226			99.45
G10	LC761227			99.64
G11	LC761228			99.64
G12	LC761229			99.70
SA01	LC761230			99.50
SA02	LC761231			99.44
SA03	LC761232			99.88
SA04	LC761233	*Xanthoparmelia conspersa*	AF117992	99.76
SA05	LC761234			99.58
SA06	LC761235			98.81
SA07	LC761236	*Crocodia aurata*	KY070338	98.58
SA08	LC761237			99.11

Table S3. List of the near-full-length algal 18S rRNA gene sequences with their accession numbers of the studied epilithic lichen samples, and the most closely related species with respective accession numbers as well as similarity values.

Sample		Closest species		
Code	Accession #	Name	Accession #	Similarity (%)
G01	LC761244	*Trebouxia aggregata*	MT901379	98.80
G02	LC761245			98.86
G03	LC761246			98.67
G04	LC761247	*Trebouxia jamesii*	Z68700	98.67
G05	LC761248			98.99
G06	LC761249			99.04
G07	LC761250			98.91
G08	LC761251			98.91
G09	LC761252	*Trebouxia* sp. TR9	KU716051	98.91
G10	LC761253			98.91
G11	LC761254			98.91
G12	LC761255			98.97
SA01	LC761256			99.16
SA02	LC761257			99.16
SA03	LC761258	*Trebouxia jamesii*	Z68700	98.50
SA04	LC761259			98.56
SA05	LC761260			98.44
SA06	LC761261	*Trebouxia aggregata*	MT901379	99.52
SA07	LC761262			99.76
SA08	LC761263	*Trebouxia* sp. SAG 2463	KM020032	99.40

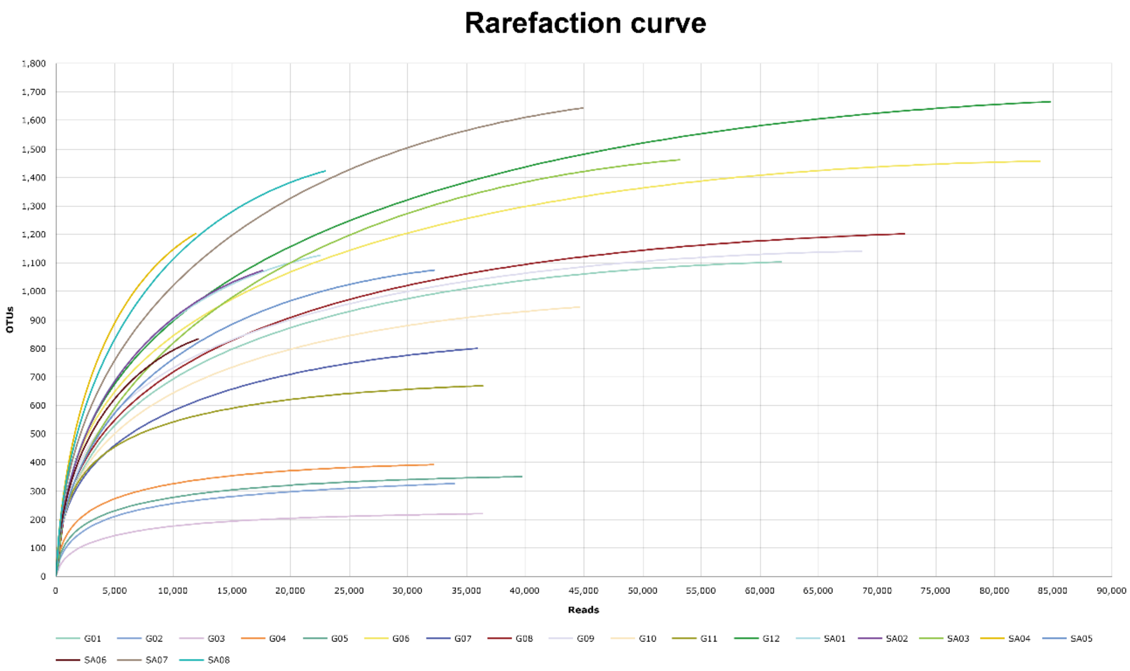

Figure S1. Rarefaction curves based on the numbers of reads and OTUs from lichen samples of twelve samples from the Venezuelan Guiana Shield (G01 to G12) and eight samples from the South African Highveld Plateau.

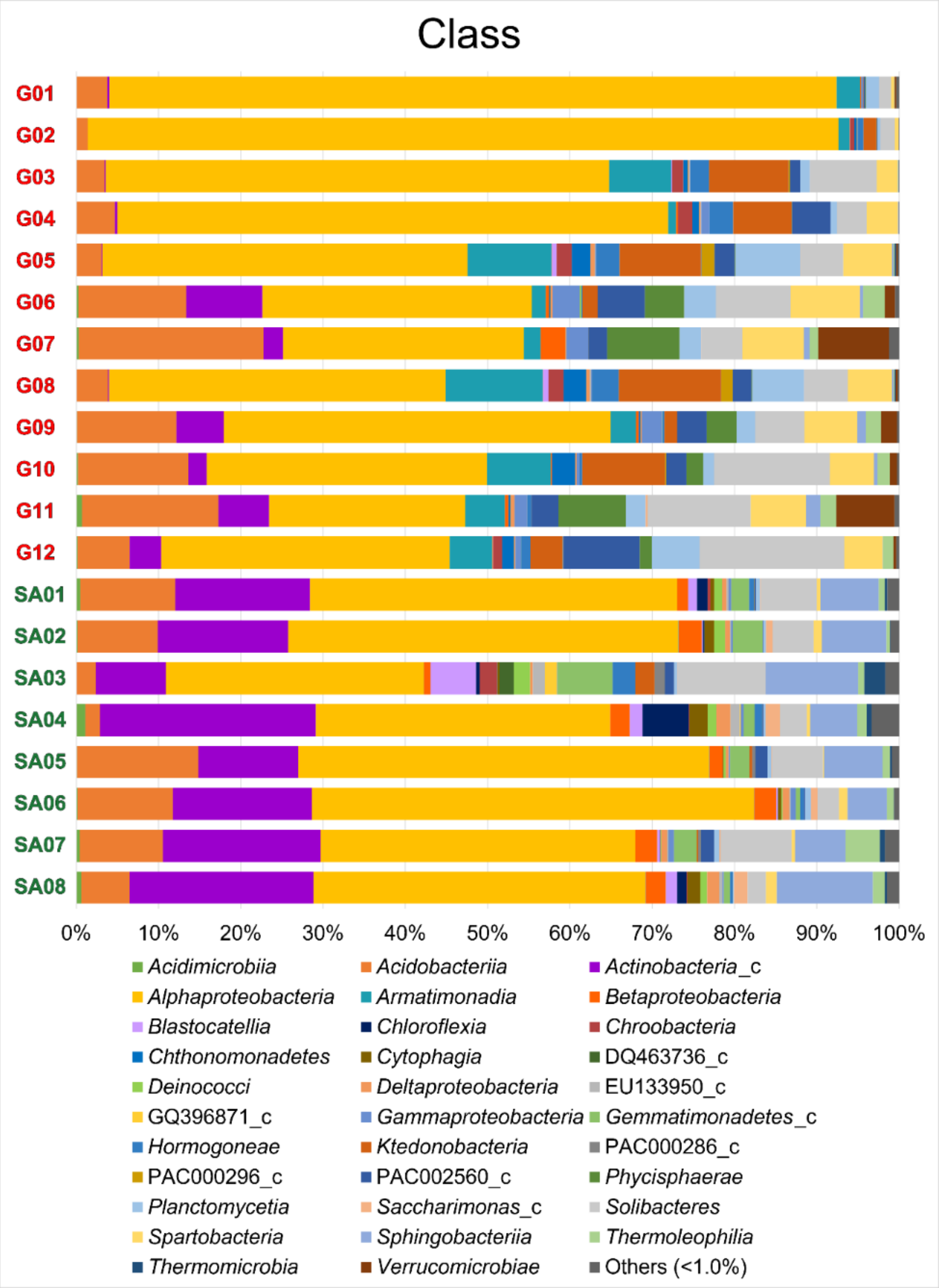

Figure S2. Bacterial class compositions of the OTUs obtained from lichen samples from the Venezuelan Guiana Shield (G01 to G12) and the South African Highveld Plateau (SA01 to SA08).

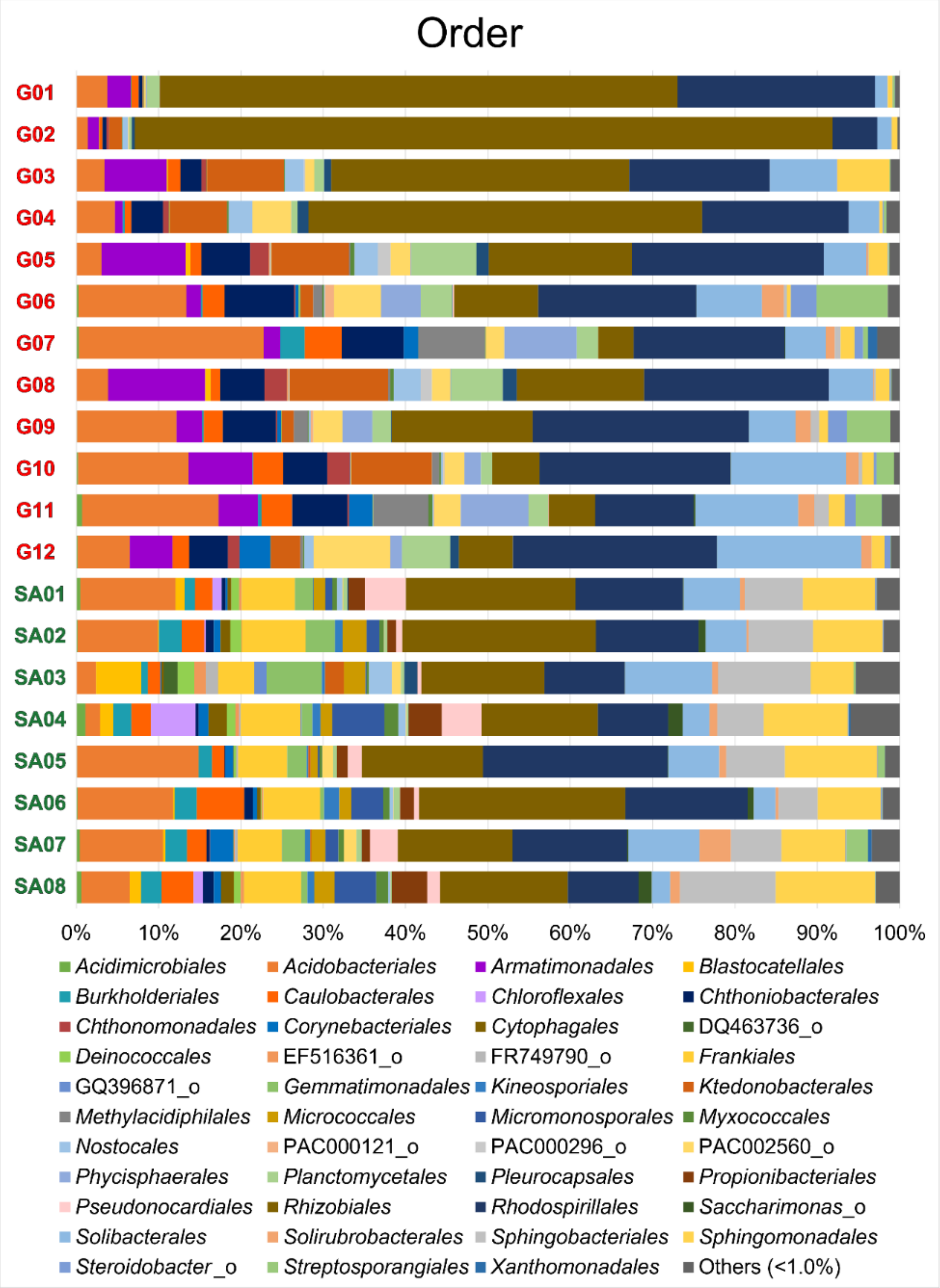

Figure S3. Bacterial order compositions of the OTUs obtained from lichen samples from the Venezuelan Guiana Shield (G01 to G12) and the South African Highveld Plateau (SA01 to SA08).

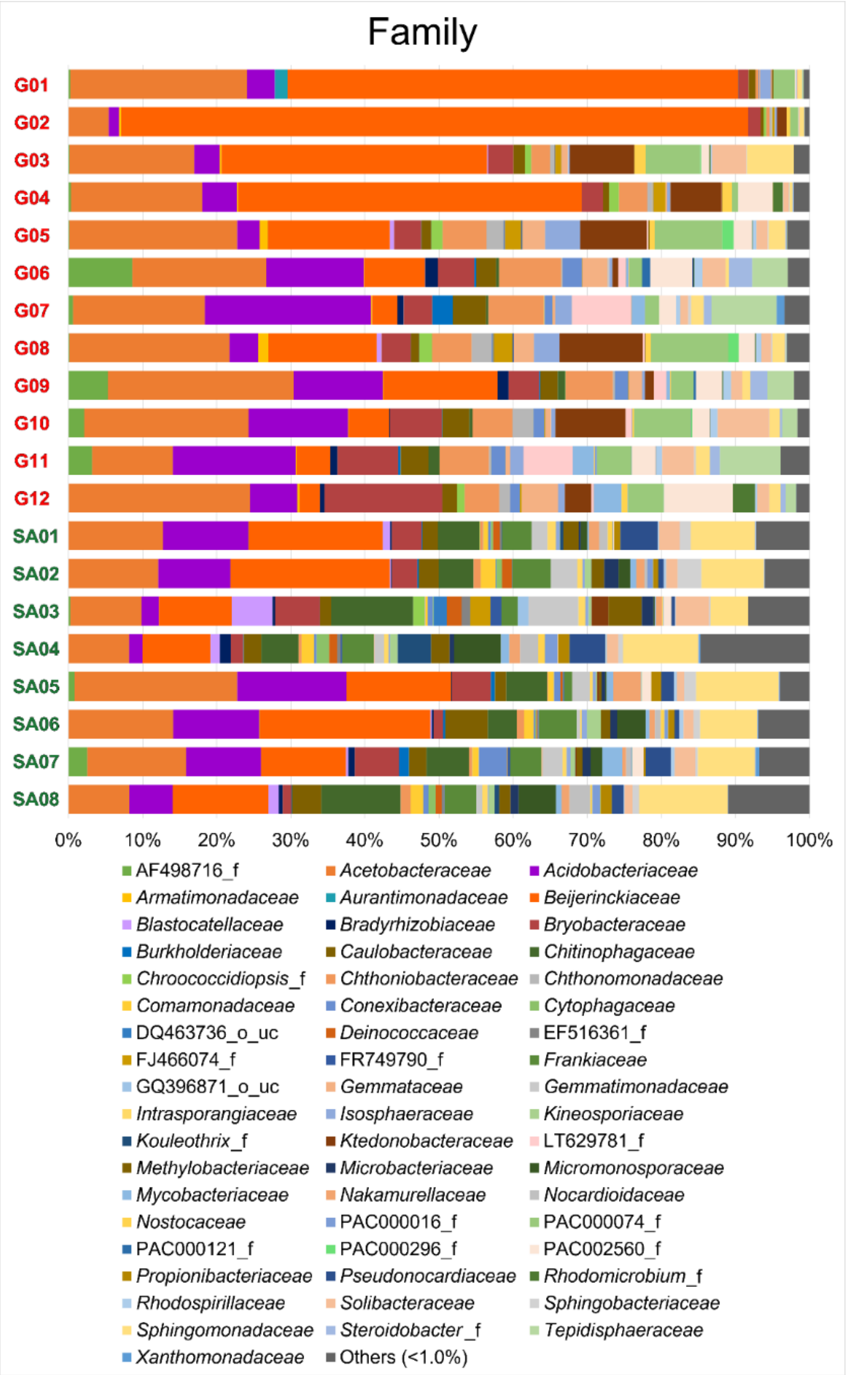

Figure S4. Bacterial family compositions of the OTUs obtained from lichen samples from the Venezuelan Guiana Shield (G01 to G12) and the South African Highveld Plateau (SA01 to SA08).

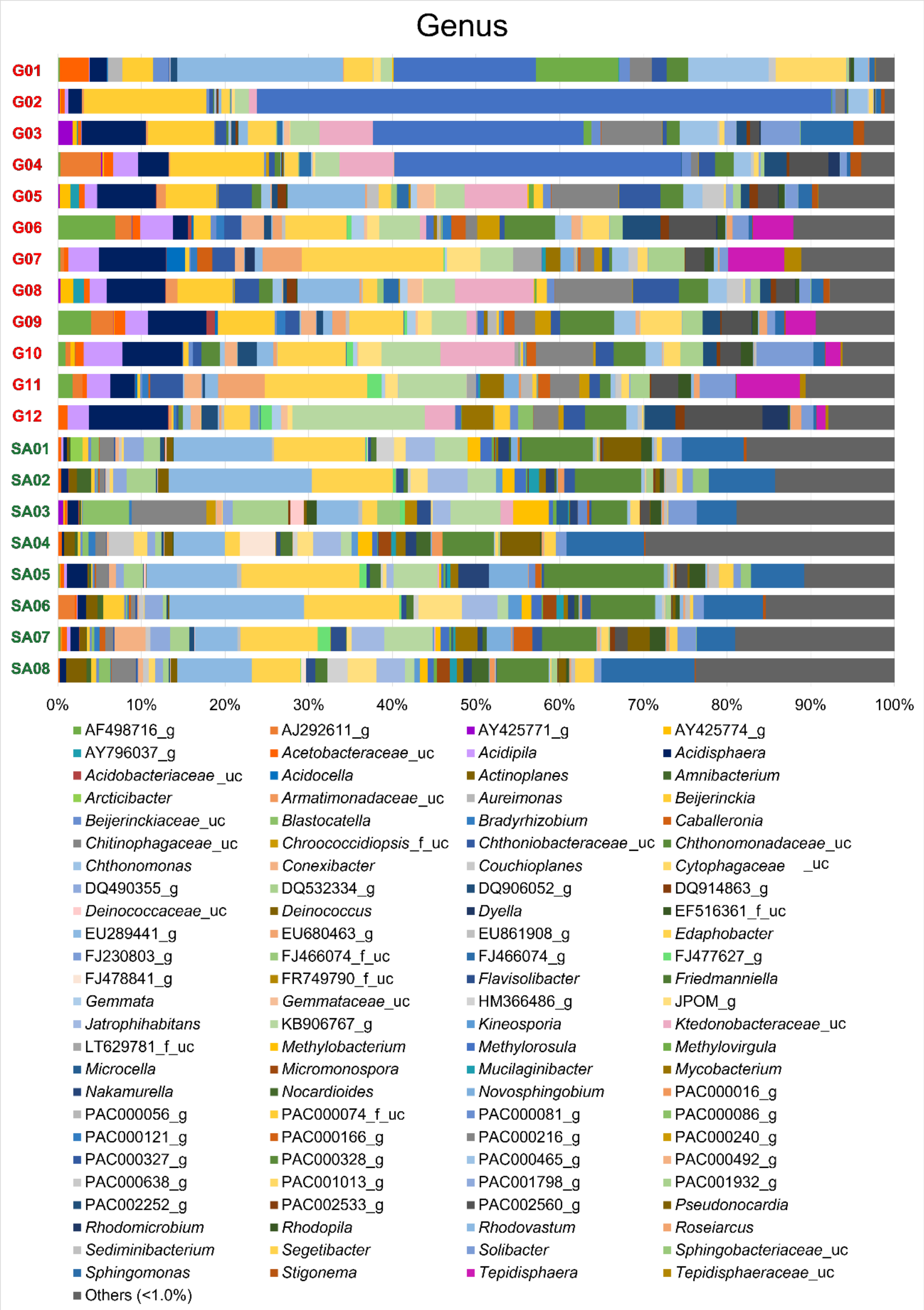

Figure S5. Bacterial genus compositions of the OTUs obtained from lichen samples from the Venezuelan Guiana Shield (G01 to G12) and the South African Highveld Plateau (SA01 to SA08).

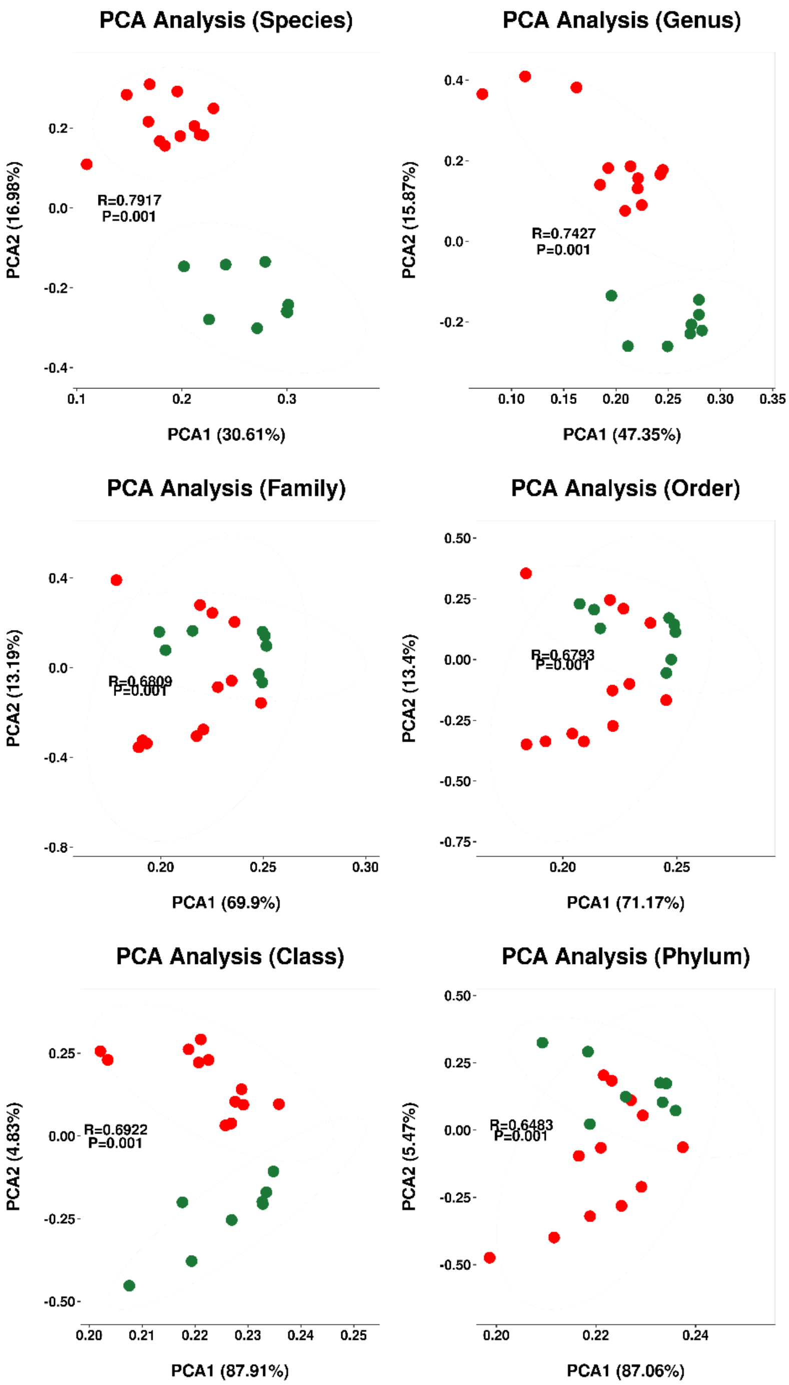

Figure S6. PCA plots of OTU-derived species, genera, families, orders, classes, and phyla of the lichens from the Venezuelan Guiana Shield (red) and South African Highveld Plateau (green).

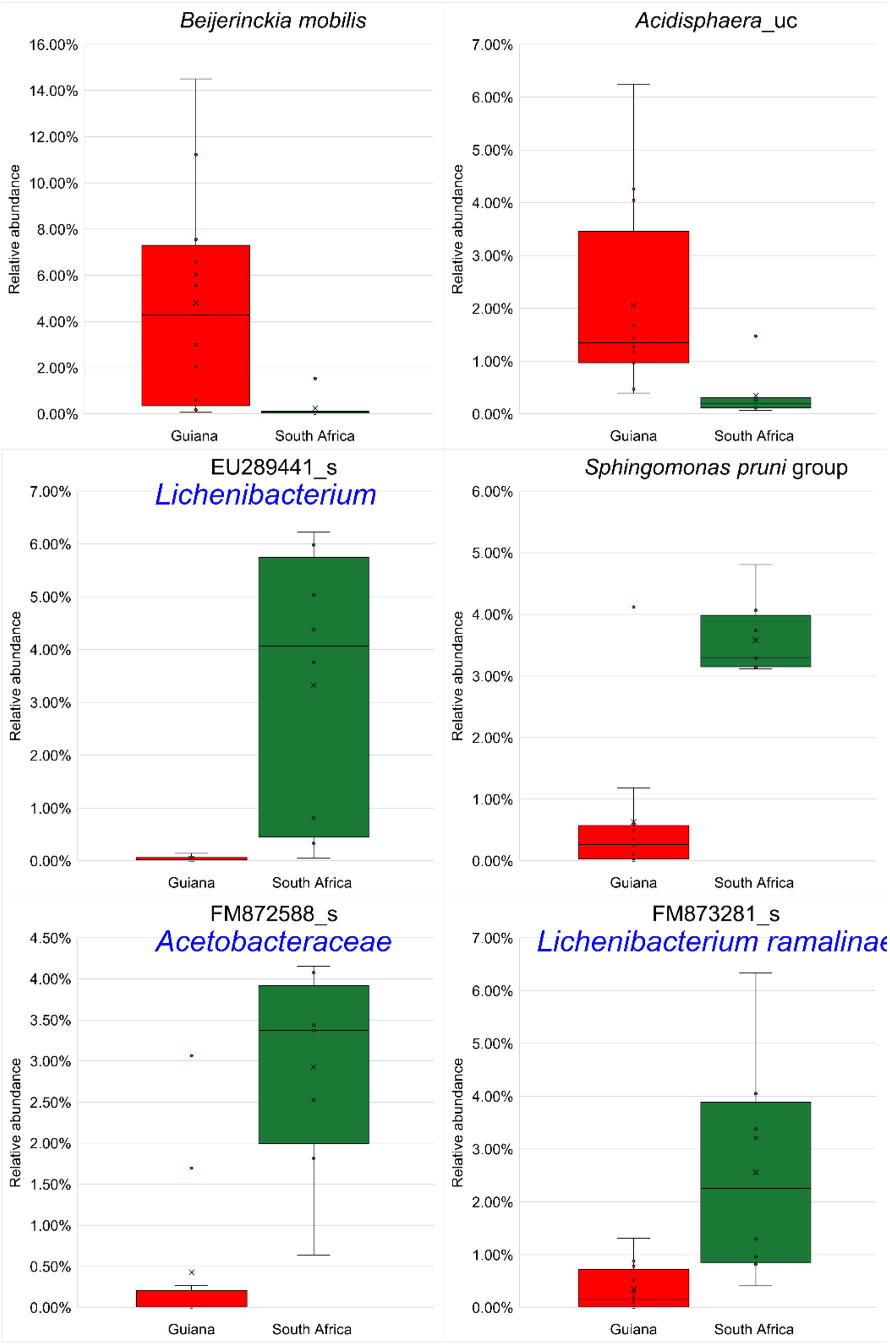

Figure S7. Significant differences ($p < 0.05$) in relative abundances of indicator OTUs of lichen samples from the Venezuelan Guiana Shield (red) and the South African Highveld Plateau (green) analyzed by ANCOM-BC. Species names and names of affiliated genera, families, orders, classes, or phyla are shown below the OTU names if applicable.

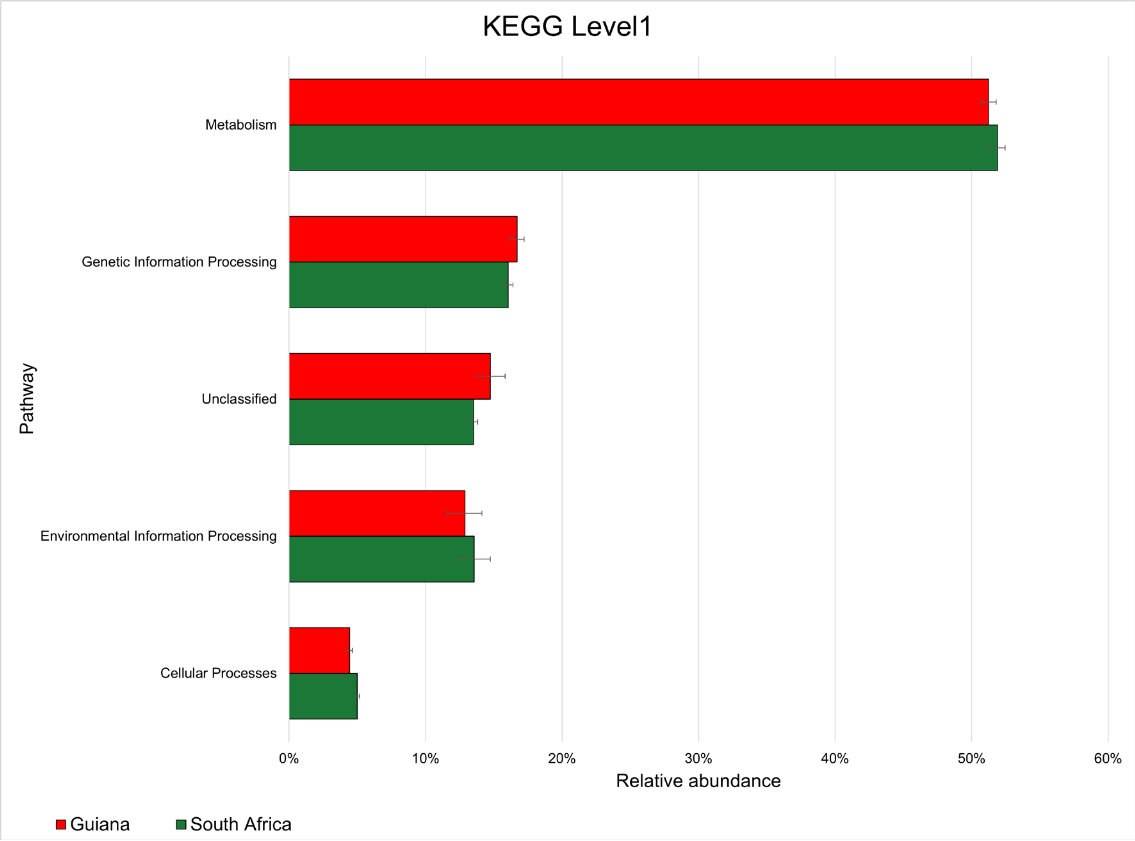

Figure S8. KEGG Level 1 metabolic pathways found in the biomarker OTUs associated with epilithic lichens from the Venezuelan Guiana Shield (red) and the South African Highveld Plateau (green).

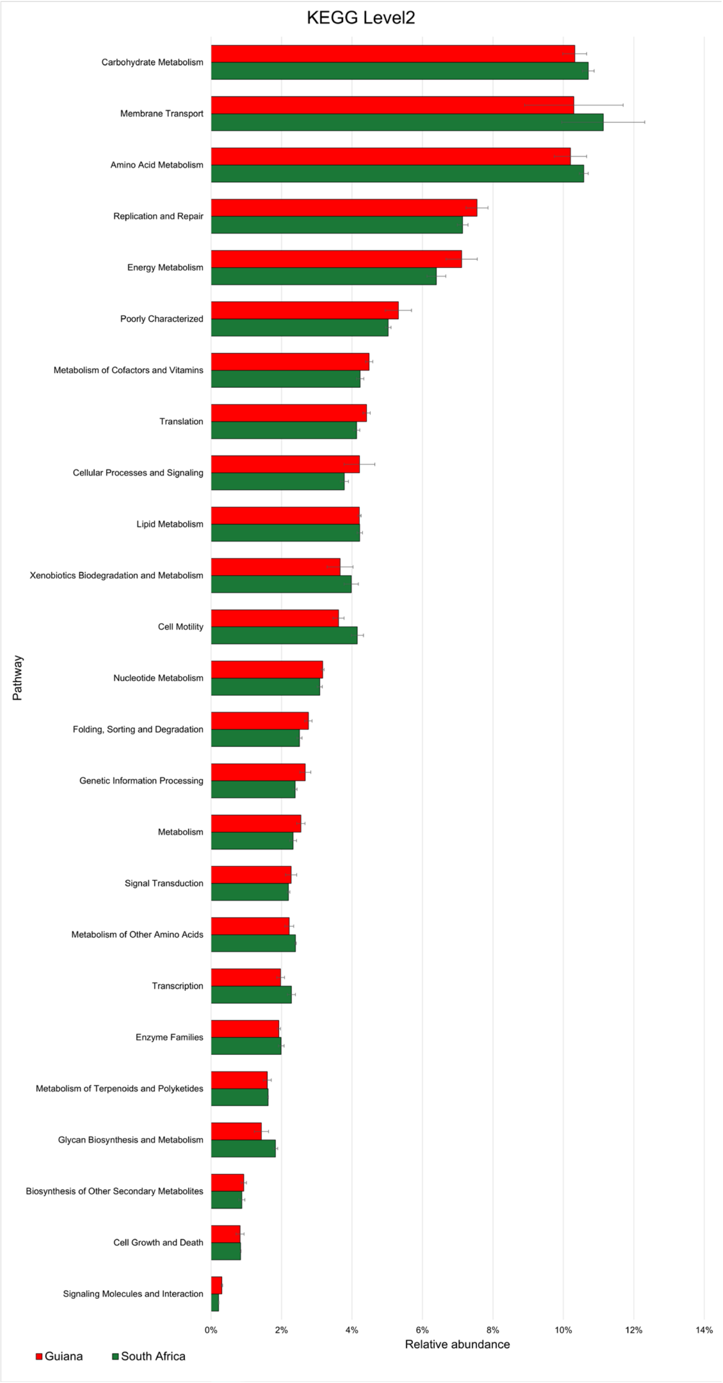

Figure S9. KEGG Level 2 metabolic pathways found in the biomarker OTUs associated with epilithic lichens from the Venezuelan Guiana Shield (red) and the South African Highveld Plateau (green).

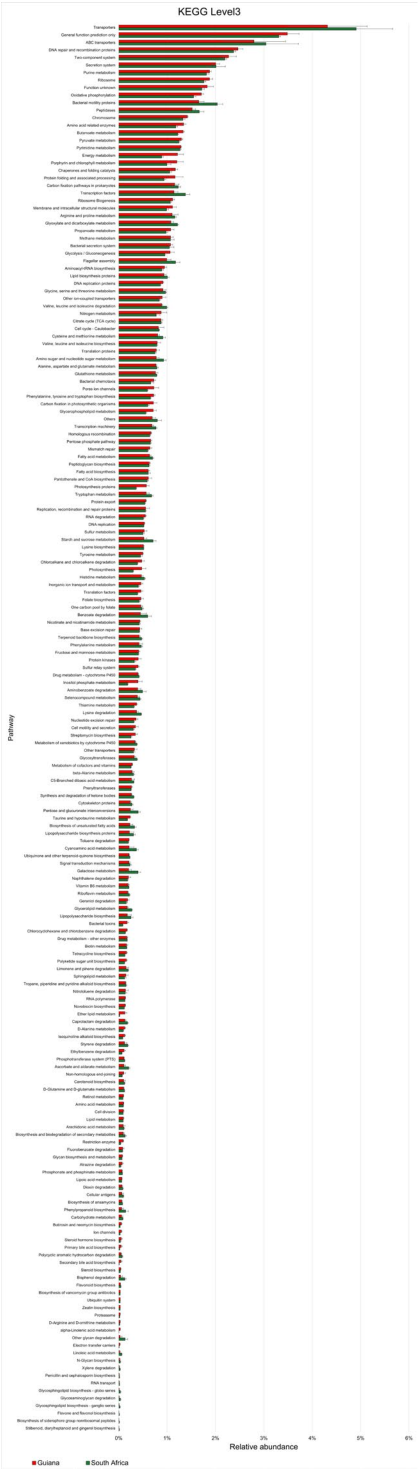

Figure S10. KEGG Level 3 metabolic pathways found in the biomarker OTUs associated with epilithic lichens from the Venezuelan Guiana Shield (red) and the South African Highveld Plateau (green).
